# Mindfulness in Psychosocial Research: An Integrative Literature Review of What is Studied and How

**DOI:** 10.5334/irsp.1000

**Published:** 2025-07-07

**Authors:** Philippine Chachignon, Emmanuelle Le Barbenchon, Lionel Dany

**Affiliations:** 1 Aix Marseille University, LPS, Aix-en Provence, France

**Keywords:** mindfulness, psychosocial perspective, social psychology, applied fields, theoretical frameworks, levels of explanation, positivist epistemology

## Abstract

In the mindfulness field, reviews of its clinical effects prevail, along with critical articles on its applications serving neoliberalism. Conversely, less is known of the psychosocial perspective on mindfulness. To address this question, knowledge needs to be gathered on the applied fields and research topics, theoretical frameworks, study designs and methodologies mobilized, main results and levels of explanation in social psychology. We conducted an integrative review of the literature in February 2022, following the Preferred Reporting Items for Systematic Reviews and Meta-analyses methodology using PsychInfo/PsychArticles. One hundred and nine papers met the inclusion criteria. Applied fields encompass well-being, daily social relationships, health and organizations. Only 21 references were embedded in theories. Forty-two percent of the theories were identified as social psychology theories. Most studies were correlational (46%) or experimental (47%) with quantitative methods. The effects of mindfulness are primarily beneficial, with a strong focus on emotion regulation and stress management at both intra- and inter-individual levels, while less attention is given to group or ideological contexts. We argue that research on mindfulness is predominantly conducted using Western, Educated, Industrialized, Rich, and Democratic samples, often without considering participants’ socio-economic backgrounds. Additionally, the prevailing psychosocial perspective on mindfulness tends to adopt a positivist epistemology, largely situated within micro-level contexts, while overlooking the broader macro-social dimensions of human experience.

## Introduction

### Conceptual Foundations of Mindfulness in Psychology

With millions of meditators worldwide (Davies et al. 2024; Simonsson et al. 2020), mindfulness has become a broad societal phenomenon, extending beyond clinical settings. As defined by medical scientist Kabat-Zinn (2003, p. 145), who originated the standardized protocol of Mindfulness-Based Stress Reduction (MBSR) in the late ’70s, mindfulness is ‘the awareness that emerges through paying attention on purpose, in the present moment, and nonjudgmentally to the unfolding of experience moment by moment’ (e.g., through the instruction of the continuous observation of one’s own breathing or thoughts). Although only Kabat-Zinn’s definition is presented here, Nilsson and Kazemi (2016) have identified up to thirty-three definitions of mindfulness. Doubtlessly, the concept is still considered to be ubiquitous and polymorph (Haddock et al. 2022) and lacking operational definition (Bishop et al. 2004). Our approach of mindfulness therefore encompasses its dominant conceptualizations as a trait, a state, or an intervention (i.e., a training) providing a wide range of formal and informal secular mindfulness meditation practices (Chems-Maarif et al. 2025; Davidson & Kaszniak 2015; Jermann et al. 2006; Kiken et al. 2015). Although scholars argue that the boundaries between secular and Buddhist mindfulness practices are rather tenuous (e.g., Brown 2016), this review focuses on mainstream and widespread secular techniques (e.g., mindful breathing) and interventions (e.g., MBSR) that are prone to affect the general population, rather than on Buddhist or spiritual currents involving specific groups such as Buddhist practitioners. Building on Bishop et al.’s (2004) conceptualization of mindfulness as both attention regulation and orientation to experience, Chems-Maarif et al. (2025) note that while this conceptualization dominates psychological studies, modern mindfulness differs from its Buddhist roots. Although the two approaches share a common component in present-centered awareness, they contrast on other elements: Buddhist mindfulness incorporates memory and remembrance, and, more essentially, ethics, while the psychological approach centers on core aspects such as bare attention, acceptance, and non-judgment (for a literature review, see Chems-Maarif et al. 2025). As conceptualized in the psychological approach, these aspects of mindfulness have served as the basis for a large body of scientific literature on the clinical effects of mindfulness in contemporary psychology. Nevertheless, it is equally important to interrogate the implications of mindfulness within social psychology—a discipline that has, so far, paid limited attention to mindfulness, despite its significant scientific potential.

### The value of mindfulness for social psychology

According to Karremans and Papies (2017), who encouraged the social psychology community to focus more on mindfulness, this field offers valuable insights into behavioral and self-related processes that can be influenced by reducing automatic responses to various stimuli. Karremans and Papies (2017) particularly emphasized these two research areas due to mindfulness processes such as non-judging and non-reactivity, decentered perspective, or awareness of one’s own responses, all considered key dimensions in contemporary approaches of mindfulness that may support improved psychosocial functioning. In addition to these domains, broader social dynamics also warrant exploration from a psychosocial perspective.

Indeed, based on its beneficial effects and the hype surrounding meditation, the outburst of Mindfulness-Based Interventions protocols (see Creswell 2017) provided in the meantime matter for heated ideological debates in various disciplinary fields (e.g., psychology, management, contemplative studies). On the one hand, systematic reviews and studies on clinical and neurobiological levels (e.g., Brown et al. 2007; Tang et al. 2015; Zhang et al. 2021) broadly document the processes and effects of mindfulness at an individual level, including its adverse effects (see Anālayo 2019; Britton et al. 2021; Farias et al. 2020). On the other hand, recently, proponents of a critical thinking (e.g., Purser 2021; Smallen 2019) have theorized and discussed the concept of ‘McMindfulness,’ a management style based on mindfulness. They pointed out the limits of a neoliberal use of mindfulness when practiced as a self-help method or to foster productivity in organizational settings (for a literature review, see Chachignon et al. 2024). Neoliberalism, as the contemporary socio-political facet of capitalism, involves not only the reactivation of the free market principles of classical liberalism, but also state-driven initiatives to implement liberalization across all spheres of society (including health and education). Additionally, neoliberalism promotes a subjectivity focused on constant self-improvement, individual regulation, competitiveness and identity-related consumption, shaping who we are through our choices and possessions (Adams et al. 2019; Bettache & Chiu 2019; Brown 2006; Lee & Zhang 2021; McGuigan 2014; Wrenn 2022). Due to its scope, social psychology is precisely well positioned to adopt this critical approach and empirically examine the connections between mindfulness and neoliberal selfhood.

### The psychosocial perspective

Interestingly, the internal processes involved in mindfulness such as present-centered awareness, bare attention, and attitudes of non-judgment and acceptance can impact the way individuals or groups think, behave and are subjectified, depending on the contexts in which they find themselves (e.g., culture and politics, socio-economic status, group membership). These processes operate at a social level, which social psychology is particularly well-suited to examine, as a ‘science establishing the continuity between individual and collective phenomena’ (Moscovici 1989, p. 409). These psychosocial phenomena can be examined through various epistemological lenses, among which two dominant and potentially complementary paradigms stand out (Jost & Kruglanski 2002). In social psychology, the positivist paradigm, which emphasizes prediction and generalization through the use of experimental methods, remains the dominant framework (Breen & Darlastone-Jones 2010). It is often seen as being in tension with social constructionism, which posits that social and psychological realities are context-dependent, shaped by culture and language, and cannot be reduced to universal laws. Constructionist approaches typically rely on non-experimental methods such as qualitative methods and discourse analysis (Jost & Kruglanski 2002). From a more critical approach, some authors (e.g., Oishi et al. 2009; Richardot 2006) have discussed the way social psychology is structured around two different, even divergent, streams. On one hand, a *psychological* social psychology addressing intra-individual processes is considered as the mainstream and dominant social psychology. On the other hand, a *sociological* social psychology tackling positional and ideological issues is considered as a critical social psychology.

Beyond these specific divergent epistemologies, Doise (1980) suggested a more integrative perspective: social psychology may as well be critical when all levels of explanations in psychology are articulated to comprehend social and psychological phenomena. The theorization consists of four levels. The first level of analysis focuses on intra-individual processes, examining ‘the mechanisms that enable the individual to organize his experience are the subject of analysis’ (Doise 1980, p. 214), such as attention regulation. The second level considers inter-interindividual interactions, analyzing how individuals relate to one another in a given situation, independent of their social positions (e.g., conflicts, aggression). The third level incorporates social positions into the analysis of psychological phenomena (e.g., intergroup bias). Finally, the fourth level examines shared beliefs, representations and norms—common ideological systems to which social groups relate (e.g., belief in a just world). In our review, we refer to these levels to emphasize the specific focus of each study. However, this does not imply that a phenomenon rigidly belongs to a single level in any context or condition.

Importantly, critical approaches to mindfulness call for an analysis of ideological and socio-normative processes at play within mindfulness in order to complement what is studied in clinical research. This aligns with the psychosocial perspective’s specific focus on phenomena. Consequently, our positioning is a sociological approach of social psychology where, as Oishi et al. (2009, p. 336) state it, we take ‘a higher vantage point and recognize macro factors as contributors to individual behaviors.’

### Research aims

The main purpose of this integrative review is to give more visibility to what constitutes the specificity or the scope of the psychosocial perspective and its epistemological foundations in the study of mindfulness. Are researchers interested in the effects of interventions depending on the contexts or situations? In behavioral outcomes? In mindfulness as practiced within groups? We aim to engage with the literature on the psychosocial aspects of mindfulness with two objectives. The first objective is to answer the question ‘What is studied?’ We will examine the populations, applied fields, research topics and findings of the studies in order to understand which domains and populations are the focus of researchers adopting a psychosocial approach. The second objective is to answer the question ‘How is it studied?’ We will examine the theoretical fields, methods, and levels of explanation in social psychology mobilized in the references. The diverse forms of psychosocial approaches in the literature on mindfulness can be characterized according to Doise’s levels of explanation in social psychology (see Doise 1980).

## Methods

### Search strategy

**Figure 1:**
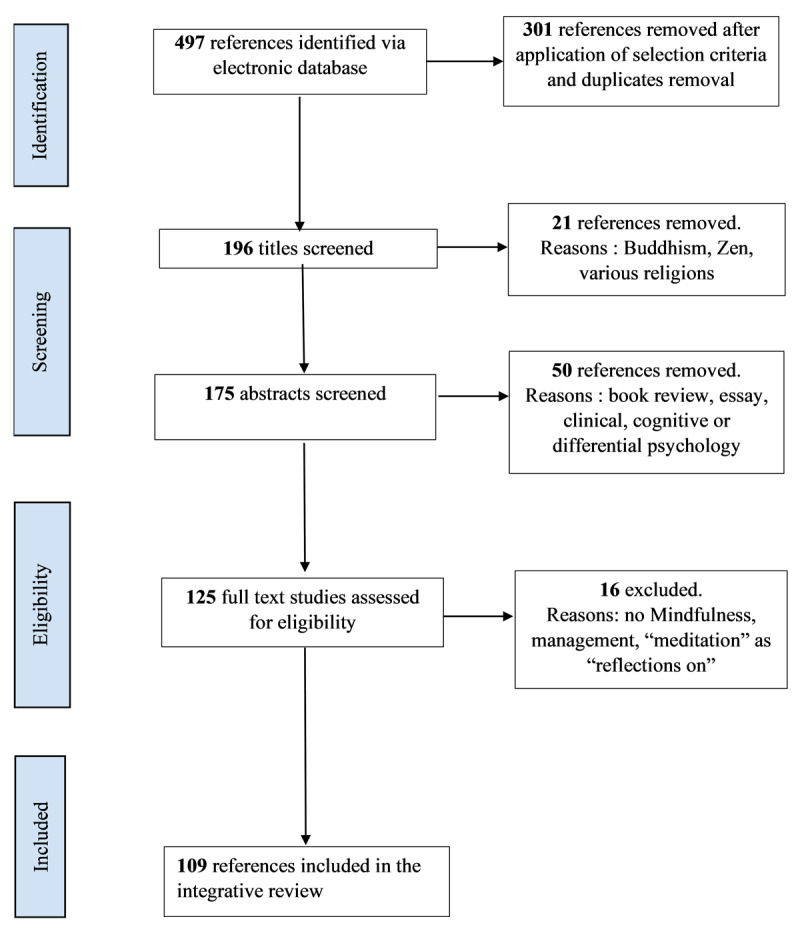
Search and inclusion/exclusion flowchart.

An integrative review of the literature was conducted in February 2022 in order to select the articles documenting psychosocial studies on mindfulness (See Figure 1 for the detailed inclusion process). Search terms ‘mindfulness OR meditation’ (subject) were combined in Boolean-type searches conducted in the PsychInfo+PsycArticles database. In addition, we selected all the classification codes that aligned with the psychosocial perspective: 2900 ‘Social Processes & Social Issues’; 3000 ‘Social Psychology’; 3020 ‘Group & Interpersonal Processes’ and 3040 ‘Social Perception & Cognition’. We chose the year 2003 as a start date because it can be considered as a starting point in the scientific upturn of mindfulness (see Kabat-Zinn 2003).

### Selection criteria

We included only studies that defined mindfulness within contemporary secular approaches in psychological science. These approaches converge on key components such as present-centeredness, attention, acceptance, and non-judgment (see Chems-Maarif et al. 2025). To ensure further conceptual consistency, we excluded studies focusing on meditation practices that were primarily religious, spiritual, or undefined. Similarly, we excluded contemplative practices that did not align with psychological approaches of mindfulness. Additionally, we excluded studies that did not fall within the psychosocial perspective as defined by the classification codes, or that explicitly identified themselves as part of another discipline. Non-peer-reviewed materials, books, and essays were also omitted to maintain academic rigor and ensure comparability across included studies. This approach ensured that our analysis focused exclusively on mindfulness as understood and operationalized in contemporary psychological science, excluding broader or unrelated uses of the term ‘meditation.’ A three-step selection process allowed the inclusion or the exclusion of the articles: first based on the title, then the abstracts and finally a full reading of the article. Along with the first author, two other scholars read the abstracts. Each of them made their own selection and explained to the others how they applied the selection criteria. Triangulation was conducted to minimize selection bias (see Thurmond 2001). The first author read thoroughly the studies matching the eligibility criteria. A PRISMA flowchart is provided in Figure 1.

### Data synthesis

Six types of data were extracted: 1) general characteristics: reference, author(s) and origin, year of publication, sample sizes, nature of the samples, type of study, methodology and operationalization of mindfulness; 2) applied fields; 3) theories; 4) levels of explanation in social psychology and 5) results. The applied fields criterion was designed as an indicator of the practical implications of mindfulness research. Specifically, it served to determine whether the psychosocial perspective considers mindfulness as a health-related issue, as clinical psychology does, or approaches it through other applied fields beyond therapeutic and behavioral processes. The theoretical foundations criterion ensured that the review captured the conceptual integration (or lack thereof) between mindfulness and core social psychology frameworks, with the levels of explanation criterion serving as an additional methodological and theoretical indicator. In order to classify the articles into the levels of explanation, we conducted a triangulation where PC and ELB separately coded the 109 selected studies and then compared and revised their coding to reach an agreement that LD, an expert in qualitative studies, verified and arbitrated when disagreement occurred. As a deductive analysis based on a theoretical framework (i.e., the levels of explanation), our approach is distinct from an inductive approach and closer in nature to a quantitative approach (see Soiferman 2010) making it less prone to subjectivity.

## Results

### General characteristics

Table 2^1^ presents the characteristics of the 109 selected articles (from 2003 to 2021) included into the review.

The majority (56%) of the references originated from North America (6% from Canada). Thirty percent of the articles were from Europe (e.g., United Kingdom, Netherlands), 9% were from the Pacific Ocean (Australia and New-Zealand) and the rest of the references (5%) were from diverse countries (e.g., China, Nigeria).

Eighty-five percent of the 109 were empirical articles (for a total of 153 studies). The rest of the references were theoretical articles (including literature reviews). Among the empirical articles, the largest sample consisted of 4,139 participants and was a scale validation study. The smallest sample consisted of 15 participants and was a grounded theory research. The participants were mostly undergraduate students (49% of the studies). The second largest category of population was the general population (21% of the studies). Thirteen percent of the studies consisted of couples and 13% comprised various social groups (e.g., sexual minorities, employees). Ethnicity was reported for 57 samples with 66.91% of the participants identified as White, Caucasians, or European. Income level, considered as a criterion for socio economic status, was reported in six studies. Eventually, only 4% of the studies included meditators. Forty-six percent of the studies were correlational (e.g., online surveys). Forty-seven percent were experimental studies (e.g., laboratory-based experiments). The rest (7%) was either quasi-experimental, using other methods or meta-analysis. The methodologies used were almost exclusively quantitative ones except for four articles: two of them used qualitative methods and two others used mixed methods. Sixty-three percent of the articles engaged with trait mindfulness, 21% with the practice (e.g., brief mindfulness inductions), 11% with the state, and 2% with both state and trait. Three percent of the references were scale reviews.

### Applied fields

Thirty-eight percent of the articles dealt with day-to-day social interactions (e.g., prosocial behaviors). Thirty-two percent of the references focused on well-being (e.g., flourishing, life satisfaction). Fifty-four percent of these studies focused on romantic relationships. Seventeen percent of the references were centered on health (e.g., diagnostic, substance use). Eight percent of the articles focused on organizational contexts (e.g., employees, coaches). The rest of the studies (5%) were interested either in ecology, education, finance or sports. These applied fields included various topics of research that formed one of the main interests of the present review.

### Theories

As displayed in Table 1, 21 references (19%) were embedded in theories. Forty-two percent of the theories could be identified as social psychology theories. The majority of the theories (58%) originated, indeed, in other disciplinary fields (e.g., psychiatry, clinical psychology, behavioral sciences). Yet, within social psychology, as outlined by Van Lange et al. (2011), several key theories are considered essential, including Cognitive Dissonance Theory, Social Representations Theory, Social Identity Theory, Social Categorization Theory, Social Comparison Theory, System-justification Theory, or Terror Management Theory.

**Table 1:** Exhaustive list of the theories present in the analyzed articles.


THEORIES IN SOCIAL PSYCHOLOGY	TIMES CITED	THEORIES IN OTHER DISCIPLINARY FIELDS	TIMES CITED

The Self-determination Theory (Ryan & Deci 2000)	3	The Attachment Theory (Bowlby 1980)	2

The Self-efficacy Theory (Bandura 1986)	2	The Schema Theory (Young et al. 2003)	2

The Interdependence theory (Kelley & Thibaut 1978)	1	The Broaden-and-build Theory of Positive Emotions (Fredrickson 1998)	1

The Moral Foundations Theory (Graham et al. 2013)	1	The Coping Competence Theory (Moreland & Dumas 2008)	1

The Social Dominance Theory (Sidanius & Pratto 1999)	1	The Marital Discord Theory (Beach et al. 1990)	1

The Action Identification Theory (Vallacher & Wegner 1985; 1987)	1	The Stress Generation Theory (Hammen 1991)	1

		The Family Systems Theory (Bowen 1978)	1

		The Family Stress Theory (Boss 2002)	1

		Cues Filtered-out Theories (Culnan & Markus 1987)	1

		The Dynamic Systems Theory*	1


Note. *No reference mentioned in the article.

### Levels of explanation in social psychology and results of the studies

The classification corresponds to the levels of explanation reached in the totality of the 109 included articles. Twenty-four percent of the references offered an intra-individual level, 54% an inter-individual level, 19% a social position level (ingroup and inter-group outcomes) and 3% an ideological/normative level (i.e., organizational level and critical articles).

In addition, the results of the empirical articles were classified according to the type of outcomes and direction (positive or negative) of the causal and correlational relationships between mindfulness and the outcome measures. The mediating and moderating effects were also included.

#### Intra-individual outcomes

From a causal perspective, mindfulness had an increasing effect on mood and positive affects or emotions (Fredrickson et al. 2008; Hsu & Forestell 2021), well-being (Jiga et al. 2019), help seeking attitudes (Oluyinka 2011), higher introspection (Nyklíček 2020) and motivation (Smyth & Milyavskaya 2021). Mindfulness had a decreasing effect on negative emotions or distress (Campbell & Pakenham 2022), negativity bias (Kiken & Shook 2011) and stress (Parsons et al. 2017) and had no effects on emotional awareness (Nyklíček 2020).

From a correlational perspective, mindfulness was positively associated with better flourishing and coping (Akin & Akin 2015), mood and positive affects or emotions (Brooks et al. 2019), living fully (Dozois 2018), satisfaction of autonomy and competence (Elphinstone et al. 2021), sustainable consumption behaviors (Geiger et al. 2019), well-being (Jacob et al. 2009), spiritual well-being (Lu et al. 2022), self-awareness and regulation (Kinsler 2014), adolescent quality of life (Lo 2021), tolerance of painful emotions (Sell 2008), awareness of financial literacy (Smith et al. 2016), equanimity (Weber 2021), and coping strategies (Womack & Sloan 2017). Mindfulness was negatively associated with ruminations (Blanke et al. 2020), emotional exhaustion (Li et al. 2017) and stress (Li et al. 2019; Witherspoon & Theodore 2021).

#### Inter-individual outcomes

Mindfulness had an increasing effect on compassion (Bankard 2015), mood regulation between partners (May et al. 2020), relationship quality or satisfaction (Langer et al. 2012), partner well-being (Laurent et al. 2016), prosocial or reparative behaviors (Dou et al. 2018; Hafenbrack et al. 2022; Poulin et al. 2021), persuasion (Gamian-Wilk et al. 2018), optimism toward transgressors (Koopmann-Holm et al. 2020), charitable giving (Orazi et al. 2021) and performance in negotiation (Reb & Narayanan 2014). Mindfulness had a decreasing effect on anger in conflicts (Barnes et al. 2007), stress during conflicts (Kimmes et al. 2018), performing interdependent tasks (Grapendorf et al. 2017), hurt feelings (DeClerck & Holtzman 2018), revenge seeking (Jeter & Brannon 2017), partners’ negative affect (May et al. 2020), punishment when being victims (McGill & Adler-Baeder 2020), dispositional attribution when perceiving anger (Pinazo & Vazquez 2014), and hostile attributions (Schans et al. 2020).

Mindfulness was positively associated with mood regulation between partners (Iida & Shapiro 2019), partner acceptance (Kappen et al. 2018), empathic perspective taking (Karremans et al. 2020), awareness in resolution of conflict (Horton-Deutsch & Horton 2003), empathy and social cognition (Campos et al. 2019), relationship quality or satisfaction (Cox et al. 2020; Gesell et al. 2020; Iida & Shapiro 2017; Karremans et al. 2017; Khaddouma et al. 2015; Khaddouma & Gordon 2018; Lenger et al. 2019; Maher & Cordova 2019; McGill & Adler-Baeder 2020; Simou & Moraitou 2018; Skoranski et al. 2019), friendship quality (Pratscher et al. 2018), sexual outcomes (Leavitt et al. 2021), prosocial or reparative behaviors (Donald et al. 2019; Harvey et al. 2019), forgiveness (Johns et al. 2015), coaching abilities (Passmore 2022), punishment when being witness (McCall et al. 2014). Mindfulness was negatively associated with anger in conflicts (Beames et al. 2019), hurt feelings (Don & Algoe 2020), rejection fears and destructive behaviors (Dixon & Overall 2018), and escalation in conflict (Smyth 2012).

Mindfulness mediated the relationship between negative emotion in attachment and avoidance of one’s anxiety symptoms (Jaurequi et al. 2021), attachment insecurity and attributions (Kimmes et al. 2017) and early maladaptive schemas and interpersonal problems (Janovski et al. 2019). Mindfulness moderated the relationships between drinking and sexual aggression such that the relationship was significant only among men with low levels of mindfulness (Gallagher et al. 2010), between distress disclosure and depression symptoms such that the relationship was significant only among students with low mindfulness levels (Kahn et al. 2017), between anxious attachment and stability such that the relationship was significant only for participants with high levels of mindfulness (Saavedra et al. 2010), between disgust and social distance such that the relationship was significant only for participants with high levels of mindfulness (Reynolds et al. 2015) and between self-control and substance use such that when self-control was low, parent–child conflict was associated with low mindfulness, which in turn increased the intensity of drug-related problems (Tarantino et al. 2015).

#### Ingroup and intergroup outcomes

At the ingroup level, mindfulness had an increasing effect on perception of leadership authenticity (Dietl & Reb 2021), collective narcissism (Hase et al. 2021) and authentic leadership (Vich & Lukeš 2018), and a decreasing effect on ostracism (Ramsey & Jones 2015). Mindfulness was positively associated with better collective sportive performances (Blecharz et al. 2014) and greater attention to ostracized individuals (Jones et al. 2019). Mindfulness moderated the relationship between self-esteem and ostracism, meaning that when mindfulness was high, self-esteem had a negative effect on ostracism (Kong 2016).

At an intergroup level, mindfulness had an increasing effect on equanimity toward dissimilar others (Baumgartner & Morgan 2019) and intergroup acceptance (Pinazo & Breso 2017), and a decreasing effect on stereotypes (Djikic et al. 2008), discrimination (Lueke & Gibson 2016) and prejudice (Stell & Farsides 2016). Mindfulness had no effect on explicit prejudice (Nicol & De France 2018), nor implicit prejudice (Verhaeghen & Aikman 2020).

Mindfulness was positively associated with awareness of skin color bias (Ransom et al. 2021) and negatively associated with prejudice (Salvati & Chiorri 2021; Schimchowitsch & Rohmer 2016).

Mindfulness moderated the relationships between stress in minorities and psychological distress such that higher mindfulness attenuated the negative impact of minority stress (Witherspoon & Theodore 2021) and between self-presentation and two self-related processes (identity clarity and self-esteem), such that students with higher levels of mindfulness better managed the impact of self-presentation on their identity clarity and self-esteem (Yang et al. 2017).

#### Ideological

In one study, mindfulness was positively associated with corporate social responsibility (Sajjad & Shahbaz 2020).

#### Other

Two studies involved scale validation (Daks et al. 2021; Pirson et al. 2018). Six papers studied mindfulness as a dependent variable (Kudesia & Reina 2019; Melen et al. 2017; Mischkowski et al. 2018; Van Doesum et al. 2013, 2016, 2018).

## Discussion

The first objective was to address the question ‘what is studied’ in mindfulness from a psychosocial perspective, by discussing the populations, outcomes, and applied fields in mindfulness research. Among the variety of outcomes, much of the interest centered on emotion regulation, stress and improvement of relationships outcomes, although the review also identified concepts related to group processes. These outcomes were generally examined in the context of daily social interactions and well-being. The second objective covered the question ‘how is mindfulness studied’ focusing on the methods, concepts, and theories used. We argue that mindfulness has predominantly been studied from a positivist perspective, and we offer a reflection on what is actually considered ‘social’ in the psychosocial perspective on mindfulness.

### What is studied (and what is not)?

First, the outcomes focus primarily on emotion and stress management. Day-to-day social interactions and well-being emerge as key applied fields. Research mainly explores mindfulness’ effects on well-being, romantic relationships, and interpersonal interactions. Managing negative emotions (e.g., anxiety, stress, anger) and fostering positive feelings (e.g., trust, forgiveness) align with mindfulness’ foundational goals (Williams & Kabat-Zinn 2013). This is particularly relevant in Mindfulness-Based Cognitive Therapy, initially designed to prevent depression relapse. However, when mindfulness is applied to areas like financial literacy, leadership, or persuasion, a critical caveat arises. These fields fall under well-being, self-help, and positive psychology, which some authors (Binkley 2011; Cabanas 2018; Hyland 2017; Rimke 2020) argue are linked to neoliberalism. In this view, well-being is seen as an individual responsibility, shaped by values of free choice and will (Van Zyl et al. 2024). The near absence of intervention studies suggests that outside healthcare settings, mindfulness appears to exemplify individualization and responsibilization (Forbes 2019; Payne 2016; Reveley 2016; Stanley 2012), positioning health as a private matter. This contrasts with social psychology interventions aimed at challenging individualism and promoting collective frameworks for well-being (e.g., Cruwys et al. 2014; Jetten et al. 2017).

Second, in this review, most studies tend to rely primarily on convenience samples, composed of students or individuals from the general population, typically embedded in the usual Western, Educated, Industrialized, Rich and Democratic (WEIRD) societies framework (Henrich et al. 2010), thus failing to mention the social contexts in which the participants were situated. As a result, key sociodemographic variables—such as socioeconomic status or ethnicity—are frequently left unspecified, reflecting either a taken-for-granted sociological homogeneity or a positivist universalism (Lee & Young 2018). This tendency, while not specific to psychosocial perspective and social psychology, indicates however that knowledge on mindfulness overly relies on (and benefits to) the understanding of WEIRD participants. In contrast, a large portion of mindfulness experiences worldwide are rooted in non-WEIRD societies, for instance in Oriental and Eastern spiritualities such as Buddhist, Hindu or Sufi traditions (Dildar et al. 2023). Moreover, trait mindfulness is supposed to be measurable across a larger variety of human beings. Another point is that, even when reported, ethnicity or income were not subjected to specific statistical tests to assess potential moderations. Indeed, a body of research advocates for considering diversity to advance the psychosocial understanding of mindfulness. This includes broadening dissemination to other audiences, particularly non-White and low socio-economic status groups, who experience higher rates of mental health conditions compared to the general population (Lorant et al. 2003; Muntaner et al. 2004) and examining possible disparities in terms of efficiency according to racial and socio-economic background, to promote the reduction of health inequalities (Foale et al. 2024). In addition, although meditators could represent a population of great interest from a psychosocial perspective—given their specific self-construals, attitudes, beliefs, or worldviews (Vieten et al. 2018), as well as the neurological changes associated with long-term practice (see Calderone et al. 2024)—studies focusing on experienced meditators remain scarce. Moreover, the choice of populations is rarely justified theoretically or empirically, despite existing literature showing that age (Shook et al. 2017), gender (Kang et al. 2018), and race or socioeconomic status (Waldron et al. 2018) influence mindfulness outcomes. This contributes to the invisibilization of crucial contextual variables (Reddy & Amer 2023).

### How is mindfulness studied?

A majority of the selected articles are correlational or experimental, with few focusing on long-term meditators. When theory-driven, studies draw on frameworks from social psychology and other disciplines, reflecting mindfulness’ clinical origins (Karremans & Papies 2017). As a result, research primarily explores interpersonal concepts like relationship quality and emotions, and only marginally examines intergroup dynamics such as prejudice or discrimination. The dominant levels of analysis are intra-individual and inter-individual (Doise 1980), while social positional and ideological levels remain underexplored. This trend highlights a broader focus within the psychosocial perspective on positivist methodologies, prioritizing objectivity over context and subjectivity (Teo 2018). Quantitative approaches dominate, with little use of qualitative or mixed methods.

Our findings align with Karremans & Papies’ (2017) psychosocial perspective, though we argue that the ‘social’ in these studies is often understood through micro-contexts rather than macro-contexts, abstracting from broader social realities. This abstraction from social and material contexts may reflect the influence of neoliberal ideology (Adams et al. 2019), where individuals are viewed as isolated from systemic inequalities and power structures. Mindfulness is often framed as a tool for individual achievement rather than a means to address systemic issues or foster community-based interventions. While some studies engage marginalized populations, they typically focus on helping individuals cope with their circumstances rather than challenging the structural violence they face. Intergroup dynamics, central to social psychology, remain largely underexplored, reducing the ‘social’ to interpersonal interactions and missing the broader social dimensions that could enrich the psychosocial study of mindfulness. This trend observed in the present review lends support to critiques of the way neoliberalism has co-opted mindfulness. As Purser (2018, p. 106) notes, ‘This tendency to downplay and minimize the social, political and economic dimension shows up in the contemporary mindfulness movement’s celebration of personal freedom, authenticity, and the emphasis on the primacy of the individual as the sole moral agent and source of authority.’

### Limitations and Future Research

First, caution must be exercised when interpreting the results, which are not to be generalized to the disciplinary fields of social psychology or behavorial sciences. Indeed, this review only included references written in English, and dating back from 2003 which does not show the entire scope of the research on mindfulness from the psychosocial perspective. On one hand, the classifications likely fall under the psychosocial domain, but this may encompass various sub-disciplines of psychology and involve collaborations with other fields. However, the most important aspect is that the review enriches the understanding of psychosocial phenomena within the context of mindfulness. Conversely, some papers might not have been included because of the selection process, such as language restrictions or databases choices. Secondly, assigning to some studies a specific level of explanation rather than another implied an interpretive effort on the part of the researchers. To ensure neutrality in this process, the three co-authors conducted separate analyses of the corpus and reached a consensus on the level of analysis assigned to each reference through a triangulation process. Another limitation lies in the epistemology of a *sociological* social psychology: if we assume that studies focusing on intra-individual levels miss part of, or do not reveal the complexity of the ‘social,’ some could argue that ‘if a higher-level explanation is more general than its lower-level competitor, there are likely to be contexts in which the causal relationships that are focal in the lower-level explanation are explanatorily important’ (Potochnik 2010, p. 68).

Suggestions for future research highlight that the psychosocial perspective on mindfulness predominantly focuses on well-being (e.g., life satisfaction) and stress at both intra-individual and inter-individual levels (e.g., romantic relationships), while other health issues and intervention studies are underexplored. To address this, we suggest that social psychologists further investigate social identity and inter-group processes using frameworks like Social Identity Theory (Tajfel & Turner 1986), Social Dominance Theory (Sidanius & Pratto 1999), and System Justification Theory (Jost & Banaji 1994). These frameworks are valuable for intervention research, particularly in group settings where ingroup and out-group dynamics emerge. They also support examining the collective effects of mindfulness, such as reducing sexism and racial prejudice, as advocated by Berryman (2024). Additionally, they provide a foundation for exploring ethical or wisdom-based MBIs aimed at fostering compassion and interconnectedness (Furnell et al. 2024).

A promising avenue for research is integrating mindfulness into a psychosocial clinic (Furtos 2015), recognizing the social origins of certain sufferings, such as precarity or occupational stress. These frameworks can ground mindfulness in ethical principles, seen as a foundation for compassionate action (Brazier 2018). On an ideological level, we suggest developing studies based on beliefs and social representations theories (Jost et al. 2008), to examine how beliefs about mindfulness shape its use and effects, such as how physicians refer patients to MBIs. Incorporating qualitative methodologies, such as focus groups or semi-structured interviews, would complement quantitative approaches in exploring the co-construction of reality in mindfulness research and applications.

## Footnotes

Table 2 is available at https://doi.org/10.6084/m9.figshare.21972776.
